# “I really can’t imagine anything that could’ve been more easy”: patient perspectives on low-threshold peer-facilitated telemedicine hepatitis C treatment for people who use drugs

**DOI:** 10.1186/s13722-026-00704-z

**Published:** 2026-07-23

**Authors:** Kim Hoffman, Gillian Leichtling, Sarah Shin, Andrew Seaman, Tonhi Gailey, Hunter C. Spencer, P. Todd Korthuis

**Affiliations:** 1 Division of General Internal Medicine & Geriatrics, Section of Addiction Medicine, Department of Medicine, Oregon Health & Science University, Portland, OR USA; 2 Comagine Health, OR Portland, USA; 3 Oregon Health & Science University, Portland, OR USA; 4 Division of General Internal Medicine & Geriatrics, Department of Medicine, Oregon Health & Science University, Portland, OR USA

## Abstract

**Background:**

Injection drug use among nonurban people has fueled sharp rises in Hepatitis C (HCV) infections. Innovative treatment models are needed that circumvent healthcare system barriers for people who use drugs (PWUD), particularly in rural areas. The Oregon HOPE TeleHCV study randomized PWUD living with HCV in rural Oregon to peer-facilitated and streamlined telemedicine HCV treatment (Peer TeleHCV) versus enhanced usual care (EUC) and assessed sustained virologic response at 12 weeks post treatment (SVR12). Peer Support Specialists (peers) conducted HCV screening in the community, facilitated pretreatment evaluation and linkage to telemedicine HCV treatment clinicians, and supported participants in HCV medication adherence. A qualitative investigation queried patients about their experiences with the implementation of the model.

**Methods:**

This qualitative study explored patient experiences with the Peer TeleHCV model in rural Oregon. We conducted semi-structured interviews with 34 participants purposively sampled from a larger randomized trial comparing Peer TeleHCV with enhanced usual care. The qualitative team analyzed transcripts using a thematic analysis framework combining inductive and deductive coding.Results. Participants described the model as accessible, supportive, and low barrier. We identified three major themes: 1) Staff approach and values facilitated engagement in care; 2) Convenience and flexibility made care accessible, and 3) Support addressing needs helped overcome barriers to care.

**Conclusion:**

Findings highlight how community-based peers, integrated telemedicine, and flexible model design together reduce structural barriers to HCV care for rural PWUD. Adapting peer-facilitated telemedicine models can inform broader implementation of low-threshold, equitable approaches for substance use disorders and infectious disease treatment.

## Introduction

A growing evidence base supports the need for low-threshold community-based models to reach people who use drugs (PWUD) outside of traditional care, including through mobile units, walk-in clinics, harm reduction programs, and telemedicine models [2, 27, 42]. Low-threshold methadone maintenance treatment is well established internationally, including through mobile and community settings. A strong evidence base supports low-threshold methadone treatment [23, 29, 30], an approach that includes elimination of access and design barriers [22]. In the United States, regulatory barriers limit access to low-threshold methadone treatment, though advocates have worked to expand low-threshold approaches for both methadone and buprenorphine treatment. (Jakubowski and Fox, [21] defined guiding principles of low-threshold buprenorphine treatment, including same-day treatment entry, a harm reduction approach, flexibility, and availability in non-traditional settings. Increasing variety in formulation options (e.g., long-acting injectables) and community settings will necessitate refinement in these principles.

Hepatitis C virus (HCV) treatment for PWUD is undergoing a similar shift to defining aspects of low-threshold models, including flexible access (e.g., walk-in or telemedicine), point-of-care testing, peer specialist support, nonjudgmental providers, addressing social needs, and care coordination [11]. HCV disproportionately affects PWUD and can be cured by effective but underutilized medications. Curing HCV can facilitate substance use harm reduction and improve self-efficacy and well-being [39, 44]. The challenges PWUD experience in accessing treatment are similar for both HCV and OUD care. These include competing priorities and unmet social needs, transportation barriers, limited provider availability, stigmatizing experiences with clinicians, and distrust of the health care system [1, 12]. The same low-threshold solutions, including telemedicine, can ease access to treatment for both HCV and OUD [9]. Telemedicine improves access to care and can be particularly useful in rural areas, which face clinician shortages [26, 28]. An innovative adaptation for HCV treatment uses staff-facilitated telemedicine, shown to effectively overcome digital and technology barriers for PWUD [16, 41, 43]. The approach holds promise for expansion of medications for OUD (MOUD) and other forms of care for PWUD.

Our Oregon-based model adds further innovation by integrating community-based peer support specialists (peers) to engage PWUD outside of care and facilitate telemedicine for hepatitis C. Peers in the model are state-certified peer recovery support specialists with lived experience of substance use disorder and recovery who are employed by community-based harm reduction and peer-run organizations, trained in outreach, motivational support, and navigation of medical and social services. Peers were instrumental in identifying the need for this model and active collaborators in model design and in refinement after launch, joining weekly study team meetings focused on process improvement. The model addresses rural challenges, including limited availability of syringe service and opioid treatment programs to support case manager-facilitated telemedicine. Peers play an increasingly important role in addiction treatment and services to support harm reduction and access to care [32, 37]. Evidence shows that support services provided by peers with lived experience of drug use can assist PWUD in achieving goals and minimizing harms related to drug use [4, 38]. Integration of peers in telemedicine treatment for HCV and OUD can expand interventions to rural areas and facilitate access for people who are disengaged from services or in areas with limited service availability.

In Oregon’s peer-assisted telemedicine hepatitis C treatment model, peers employed by community-based organizations engage PWUD through street and encampment outreach and outreach to harm reduction programs, social service organizations, and other community partners [19]. Peers facilitate HCV testing and linkage to telemedicine HCV clinician encounters through the state’s academic medical center, and support participants in HCV medication initiation and adherence and completion of labs at 12 weeks after HCV treatment. The model uses streamlined confirmatory testing procedures in which peers use a tailored standing order lab form, provide transportation to the lab, and work with OHSU staff on health insurance enrollment if needed. A clinical coordinator communicates with peers about patient results and is responsible for tracking and communicating with peers about the patient’s progress through the HCV care cascade. When participants are ready to meet with the clinician to discuss treatment, peers contact the clinical coordinator to request an on-demand telemedicine visit, which the peer facilitates wherever the peer and participant are interfacing, including peer organization offices and in the community (e.g. homes, cars, campsites, parks). Clinicians typically provide same-day access for the visit to discuss and initiate prescriptions, with weekday availability from 12:30–7:00 p.m. Patients choose the location of medication delivery, whether to a secure medication locker at the peer organization office, a residence, or in some instances a local pharmacy; and peers assist with transportation or delivery as needed. The clinical pharmacist conducts patient check-ins after medication initiation and to facilitate medication refill, enabled by peer outreach as needed. Peers provide ongoing peer support services to participants, maintaining communication according to participant needs and preferences, including phone, text, street/encampment outreach, home visits, and other means. Clinicians provide on-demand access to same-day telemedicine follow-up visits to address patient concerns or questions. Previous publications describe the model in greater detail, including clinical approach to minimize invasive testing and streamline treatment delivery [17, 36].

The Peer TeleHCV trial tested the model by randomizing 203 PWUD living with HCV in rural Oregon to peer-assisted and streamlined telemedicine HCV treatment (TeleHCV) versus enhanced usual care (EUC) consisting of peer referral to local care [18]. Peer activities occurred at each stage of the cascade of care: (1) case-finding and HCV antibody screening through peer outreach; (2) facilitation of confirmatory RNA testing and pretreatment laboratory work; (3) linkage to a telemedicine clinician visit; (4) facilitation of medication delivery; (5) ongoing adherence support, reminders, and check-ins during the 8–12 week treatment course; and (6) facilitation of post-treatment SVR12 laboratory work. Peers center the locus of care in the community, with linkage to remote office-based clinical personnel. The community-based peer and patient form the central dyad, with the peer facilitating communication between clinical personnel and the patient. Peers play a lead role in identification and diagnosis, using their knowledge of local community contexts and informal networks to implement community-based case finding, point-of-care HCV antibody testing where feasible, and phlebotomy facilitation. As discussed in our previous publication [20], peers are essential in treatment linkage, initiation, and completion: Peers build on the relational trust that supports engagement of people disengaged from formal care, provide motivational support to initiate HCV care, bridge technology gaps, aid in medication pickup, and conduct community-based outreach to support treatment completion. In these ways, the peer role improves accessibility and effectiveness of HCV care, when combined with responsive, on-demand, non-stigmatizing care from clinical personnel. These distinct synergistic roles improved effectiveness (treatment initiation, adherence), efficiency (same-day access, fewer missed visits), accessibility (in-community care, removal of transportation barriers), and availability (extended-hours access, mobile support).

The trial found significantly and substantially higher HCV treatment initiation and cure rates for TeleHCV than EUC [33] and significantly greater reductions in injection drug use ([35] a). A concurrent qualitative study using patient interviews described the importance of peers in providing relatable lived experience, bridging gaps in patients’ trust of the health care system, and facilitating access to needed resources. Quantitative results from the Peer TeleHCV randomized trial demonstrated substantially higher treatment initiation and cure rates in the telemedicine arm compared with enhanced usual care. Our qualitative findings help explain these effects by illustrating how peer support, rapid telemedicine access, and flexible medication delivery mechanisms made it feasible for participants to engage in and complete treatment despite significant structural barriers. Consistent with Jakubowski and Fox’s definition of low-threshold buprenorphine treatment, we use the term ‘low-threshold’ to describe models that minimize requirements for entry and continuation in care (e.g., same-day initiation, harm reduction approach, flexibility, and availability in non-traditional settings). Because these approaches aim to remove structural and logistical barriers, they are often described as ‘low-barrier’ models; in this manuscript, we use ‘low-threshold’ as an umbrella term encompassing these low-barrier features.

This qualitative study explored patient perspectives on the acceptability, perceived impact, and key features of the Peer TeleHCV model. Our aim was to understand patients’ experiences of low-threshold, peer-facilitated telemedicine for HCV treatment. Specifically, our research questions centered around: (1) How did patients experience the peer, telemedicine, testing, and medication-delivery components of the model? (2) Which features of the model supported or hindered engagement, treatment initiation, and medication adherence? (3) What model elements do patients identify as most important for replication or adaptation in other settings?” As reported here, the qualitative study provided a window into patients’ overall experiences with the multifaceted low-threshold care model that includes peer support, same-day or walk-in telemedicine clinician access, streamlined testing protocols, and medication delivery innovations. Accordingly, this qualitative study explored patient perspectives on the acceptability, perceived impact, and key features of the Peer TeleHCV model. We aimed to understand how patients experienced this low-threshold, peer-facilitated telemedicine approach to HCV treatment and how model components supported or hindered engagement, treatment initiation, and medication adherence. Quantitative results are reported elsewhere [33].

## Methods

We conducted semi-structured qualitative interviews using a QUANT-qual design that focused on understanding patient experience with the Peer TeleHCV treatment model [7]. We recruited participants using purposive sampling (*n* = 34) from the larger study sample (*N* = 203). Participants in the parent randomized trial were recruited by community-based peers conducting outreach in three rural Oregon counties (Coos, Douglas, and Josephine). Peers engaged potential participants at harm reduction programs, syringe services, encampments, and through street outreach, as well as via referral from harm reduction partners, social service organizations, and word-of-mouth among community members. There was no traditional advertising and no recruitment through health care clinicians; engagement relied on peers’ existing relationships and outreach in community settings. Study participants were stratified into two medication adherence groups (completed 50%+ or less than 50% of medication based on self-report in study surveys) and randomly selected within strata. The intent was to include participants with varied degrees of challenges to treatment. No one declined participation or ended the interview prematurely. Interviews were conducted from January - March 2023 and participants represented three rural Oregon counties. Interview participants included those randomized to TeleHCV (*n* = 22) and EUC (*n* = 12). EUC participants who did not initiate local HCV treatment within 6 months after randomization were offered TeleHCV, and 7 of 12 EUC interviewees received TeleHCV. All interviews were completed after participants’ SVR12 lab date, on average 5.5. months after. In the parent trial, EUC consisted of peer-supported referral to local community HCV treatment providers (rather than telemedicine through the OHSU TeleHCV team). EUC participants still met with peers for HCV screening, education, and referral, but were not offered facilitated same-day telemedicine, streamlined confirmatory testing through the study team, or supported medication delivery during the initial randomized phase.

For the qualitative substudy, we purposively sampled from the 203 enrolled trial participants to ensure variation across study arm (TeleHCV vs. EUC), county, gender, age, and HCV treatment trajectory (treatment initiated and completed, treatment initiated but not completed, treatment not initiated). Peers and the study coordinator approached eligible participants and described the qualitative interview; interested participants were referred to the qualitative team (KH, SS) for scheduling. We continued purposive sampling until thematic saturation was reached.

We conducted interviews from January to March 2023, and participants represented three rural Oregon counties (Coos, Douglas, and Josephine). Interview participants included those randomized to the TeleHCV model arm (*n* = 22) and EUC (*n* = 12); EUC participants who did not initiate local HCV treatment by the end of their study outcome period were offered the TeleHCV model. Of the 12 EUC participants interviewed, all but one had crossed over and received some or all of the Peer TeleHCV components by the time of interview.

The research team included experts in addiction medicine, qualitative researchers, and individuals with experience in harm reduction service delivery; the team engaged in reflexive discussions to minimize the influence of preconceived notions. Interviews were conducted via phone by trained qualitative interviewers (KH and SS) independent from intervention delivery and unknown to participants. At the start of each interview, they emphasized their independent research role, confidentiality of responses, and interest in hearing both positive and negative experiences to help reduce social desirability bias. Research was conducted in accordance with the Declaration of Helsinki and all participants provided informed consent prior to their inclusion in the study. A semi-structured interview guide explored participants’ experiences with the TeleHCV model and participants received a $40 gift card upon completing the interview. We professionally transcribed recordings and redacted all identifiable information. We monitored for thematic saturation throughout data collection and analysis and determined that no new major themes were emerging by approximately the 30th interview; the final four interviews confirmed and elaborated existing themes rather than introducing new ones, supporting adequacy of the sample size.

### Analysis

We conducted a Thematic Analysis [5] using a semantic, inductive approach to identify themes. Three members of the study team (KH, GL, SS) developed a thematic coding framework. Double-coding 10% of the sample with the established framework yielded an inter-coder agreement of 85% and remaining transcripts were coded (KH and SS) using ATLAS.ti Web (v4.6.0-2022-12-06). Following Braun and Clarke’s phases of thematic analysis, the team first engaged in repeated reading of transcripts to achieve familiarization, then generated initial codes across the dataset before iteratively grouping codes into candidate themes and subthemes. Themes were reviewed against coded extracts and the full dataset and refined through team discussions until consensus was reached on their scope and boundaries. Although only 10% of transcripts were formally double-coded, coders met regularly to review coding decisions, and challenging or atypical transcripts were discussed jointly to reduce single-coder bias. Oregon Health and Sciences University Institutional Review Board reviewed and approved the study (IRB# 20911).

## Results

Of the 34 participants, most identified as male (59%), age 30 years or above (91%), and White non-Hispanic (91%) (Table 1). We identified three major themes from the interview data including:

1. Staff approach and values facilitated engagement in care.
2. Convenience and flexibility made care accessible.
3. Support addressing needs helped overcome barriers to care.

## Theme 1: Staff approach and values facilitated engagement in care

Participants described the way providers and staff engaged with them as an important factor in the model, highlighting three areas: (a) peer encouragement and caring, (b) provider nonjudgmental care and alliance with patients, and (c) phlebotomist skill and sensitivity.

### Subtheme 1a. Peer encouragement and caring

Peers provided a sense of genuine caring for participants. Participants viewed peers as caring about them as individuals, evidenced by peers’ responsiveness, attentiveness, and persistence. One participant described their peer as an “all-in advocate” (P1207). Others shared similar experiences.

*It makes me feel like there’s actually nice people out there that actually*,* genuinely*,* care and just not try to get me through the door to the next person. Who actually took time to explain stuff. Actually*,* took time to get ahold of me. When I’m trying to get ahold of her*,* she’s real quick at it. She’s awesome. P1067*

*She was there the whole time with me every visit over the phone or whatever. She was there supporting me all along the way. […] She really cares about me a lot*,* I think*,* because she made sure that I was taken care of well. P1086*

*6051: She was always there for me*,* always helping me*,* always giving me a heads-up. I’ve blown her off a lot. I just thought she’d give up on me a long time ago*,* but she never has.*

Participants described the motivational support and encouragement provided by peers as key to their ability to continue through the steps in the process, linking this encouragement at times to the peer’s sharing of personal lived experience.

*Because there were times when I wanted to walk away. Literally*,* she came and was like*,* “Well*,* come on. It’s not going to take long. Just get in the car. Come on. I’ll take you. You won’t have to do nothing. Take a nap*,* and you’ll be back before you know it.” P233*

*She shared some of her experiences*,* I guess*,* and just told us if we kept on doing the right thing*,* we can come through the other side*,* too. I mean*,* super down to earth*,* just bad ass lady—absolutely. One of the coolest people I’ve probably ever met. P1372*

*She was really supportive*,* too. Like I said*,* we both opened up to each other. She told me her experience and everything. P6157*

Participants expressed that the presence of the peer during often challenging blood draws was calming and empowering, and that the peer helped assuage their anxieties.

*Actually*,* at first*,* I was a little nervous*,* but she made me feel comfortable. She sat right next to me the whole time. She really just made me feel super comfortable and relaxed. She just talked me through it*,* and everything went smoothly. Actually*,* it was a good experience. P4149*

### Subtheme 1b. Provider nonjudgmental care and alliance with patients

Participants shared their experience of receiving nonjudgmental care from the telemedicine clinicians, often contrasting the clinician interactions with past experiences of enacted stigma by health care providers. Participants expected to experience judgment for their drug use or hepatitis C status and found it notable that the clinicians treated them as equally deserving of care as other patients.

*I wasn’t treated like I was an addict or nothing like that. I was treated like just another human being. I didn’t feel like they talked down to me or anything like that. They were talking to me like I was very cool*,* not like I was any less than them*,* so it was pleasant. Usually*,* in like a hospital situation or most doctor situations that I’ve been in—actual face-to-face that I’ve been in—it feels like a judgmental situation. Since they know that you’re an addict*,* they start to look down on you or talk down to you. It’s like a judgmental thing. I didn’t feel any of that with this telemedicine at all. They talked to me like I was an equal. P1201*

*They just talked to me like I was a human. They didn’t talk to me like I was worthless or didn’t actually deserve it or question why I had hep C or any of that. They didn’t do none of that. They weren’t negative in any way. They were all pretty positive. Like I say*,* they were compassionate and understanding*,* and that’s a big thing. P4053*

*It was really good. I didn’t feel judged or anything. I figured maybe they might be a little judgmental or something*,* and she wasn’t. The doctor was really cool—answered all the questions I had. It was quick. It was easy. It was painless. We were done. It was good. P4195*

A sense of comfort was commonly reported, when asked to describe interactions with providers. Participants felt that their providers were informative, attentive, and listened to their concerns.

*She listened to what I had to say. She didn’t tell me what I was going to say. P6076*

*He was super nice. He just basically explained to me the routine on how to take [the medications] and things to watch out for*,* as far as side effects and stuff like that*,* but he was really cool. He made himself available for me to call any time. P4233*

*They’re just all really understanding and didn’t make me feel awkward for problems I’ve had. It was pleasant speaking with them. […] It went very smooth. Very helpful. I didn’t have to do anything. I was just cared for during the whole thing. P6051*

Clinicians formed an alliance with patients and gave them a feeling of hope and empowerment. Participants noted feeling acknowledged and “wanted” as patients.

*Yeah. He was great. It was a real good experience because he didn’t give up on me*,* because I’ve had hep C so long and I’m older. He didn’t just throw in the towel*,* and I was really happy about that. He gave me some hope that*,* even though I have the cirrhosis*,* that if I’m cured with the hep C*,* we can stop any more of the damage in my liver. P4312*

*I could tell that he was like he was proud to see such a thing. I felt like he wanted to be my doctor or whatever. P1002*

*My phone kept hanging up because of the location I was in*,* and every time*,* he called me right back. It must have been four or five times that happened*,* and he wasn’t upset or anything. He’s just really a positive physician and real optimistic. I really enjoyed that. P1255*

Participants shared that the clinicians created a sense of personal connection during the video or phone consultations. As one participant described:

*I couldn’t ask for a better doctor*,* especially through the phone. Because you’d think it’d be more impersonal*,* but I disagree with that. It was really personal*,* and it was just amazing… I’ve never had really any experiences similar to the doctor on the phone*,* not face-to-face ones. P1255*

### Subtheme 1c. Phlebotomist skill and sensitivity

Although phlebotomists were not part of the study care team, the team met with local labs prior to study launch, and peers used their experience to identify lab staff most likely to bring the skill and unbiased attitude needed. Participants reported that the lab technicians and phlebotomists were professional and skillful, despite participants self-identifying as having challenging veins. The staff’s nonjudgmental attitude contrasted with past negative experiences.

*My veins aren’t the best to always get a vein*,* and they’re very gentle and made sure not to try to poke me a bunch of times to get it. They were diligent in making sure they’d be able to get it on the first try*,* which was really pleasant that way. I’ve had a lot of experiences with phlebotomists who were not nearly as pleasant as this facility*,* so I have to give five stars. P4331*

*They were all very understanding there too*,* but I’m a very hard draw. I’m very hard to get blood from*,* so it was kind of difficult. The experience kind of sucked. The nurses were wonderful. I mean*,* they asked me*,* “I’ll stop if you want me to.” I was*,* “Well*,* let’s just keep trying.” I just wanted it done*,* but they were very understanding*,* so that part of it was really good. I mean*,* they tried to make me as comfortable as possible*,* so it was kind of nice. P4053*

*Oh*,* I was treated nicely at the lab. The first time*,* the lady was very sweet*,* very kind*,* very caring. Even though I had a panic attack*,* she waited a second. She wasn’t impatient or anything like that. She was good. It was great. P4195*

## Theme 2: Convenience and flexibility made care accessible

Three subthemes emerged related to the convenience and flexibility of the model: (1) mobile, in-the-community support, (2) rapid access to telemedicine, and (3) supported telemedicine and medication delivery.

### Subtheme 2a. Mobile, in-the-community support

The Peer TeleHCV model’s out-of-office care approach was a key benefit highlighted by participants. As most participants did not own cars and given the rurality of the study locations, peers brought telemedicine care and resources to participants as needed.

Peers provided transportation to the laboratory for blood draws. Participants felt that without this assistance, they would have had difficulty making to the lab, especially in cases where multiple visits to the lab were required due to inability to draw enough blood to run all needed tests.

*I don’t have a car. At the time*,* I was homeless*,* so I didn’t have a way to get there. [My peer] came. She picked me up*,* and she took me to the lab to get tested. She took me to the lab to get tested again. She took me everywhere I need to go. P4195*

Peers brought telemedicine to participants as needed, meeting participants in their home or other private setting, setting up in a peer organization van, or transporting participants to join telemedicine visits from the peer office. Participants described this element of the model as essential to their ability to access care.

*I didn’t have to go into the doctor’s office. I didn’t have to leave. I just had to go 15 min away from me where I stayed*,* instead of having to go all the way into [city name] and to waste the whole day doing it… P4195*

*It was a lot easier. Didn’t have to drive to town*,* didn’t have to sit in a hospital or waiting room or anything. It was a lot easier and more convenient. A lot more convenient. P4311*.

*At the time*,* I didn’t have a phone either. I was off the grid. I didn’t have a car or a phone*,* and so [my peer] was just coming and getting me. Actually*,* I made the call. I talked to the doctor in [my peer’s] van*,* off of her phone.” P4233*

*I talked to a doctor the first time in a van with [my peer]. […] It went really smooth because it’s just that the doctor was real easy to talk to*,* and [my peer] was there to help us with the technicality stuff. It was a nice experience actually. P1224*

### Subtheme 2b. Rapid access to telemedicine

The Peer TeleHCV model included a streamlined line of communication between the peers and clinical team to expedite access to telemedicine visits; in particular, initial visits to review lab results, initiate medication prescriptions, and address questions and concerns. Typically, the team provided same-day or “walk-in” access to care. Participants appreciated the convenience and ability to take advantage of their readiness and availability in the moment.

*I didn’t have to not go to work or make another appointment because of it. [My peers] came and I sat in the van. Then they punched it on the computer for me*,* and I talked to the doctor that way. It was real easy…It would be a walk-in almost…I didn’t have to wait. P1224*

*[My peer] was just like*,* “Well*,* let’s just do it right now*,* if it’s possible to get it done.” … I like to just get things taken care of and done*,* get them off my agenda or whatever. …I really can’t imagine anything that could’ve been more easy. P4331*

*Yeah*,* it only took—like I went to the needle exchange*,* sat on the computer for about 10 min. [inaudible] I was done. I got the rest of my day…I didn’t have to wait for an appointment or nothing. [My peer] just called the doctor*,* and the doctor was right there. I was like*,* “Hell yeah!” That was cool. P4195*

### Subtheme 2c. Facilitated telemedicine and medication delivery

Peers facilitated telemedicine appointments by directly coordinating with clinicians, ensuring patients had access to necessary technology, and helping them understand what to expect. Peers tailored support to each patient’s needs—whether by bringing technology to the patient, assisting with their own devices, or transporting them to the peer office for the visit—and facilitated access:

*[My peer] lined everything up to make sure that [physician] was available*,* which was awesome. It was just pretty awesome. I think that’s the biggest thing right there is having somebody like [my peer] on your side. She doesn’t want to waste your time. She wants to make it easy. Yeah. [my peer] is absolutely amazing… She*,* of course*,* let us know what to expect*,* what kinds of questions [the clinician is] going to ask. I mean*,* she just made everything super simple and easy just so we’re not thrown off guard. P1372*

Participants without a physical address to receive medications could have the medication package mailed to the peer organization to be held in secure medication lockers. The study team developed a locker protocol that ensured patient control of and access to HCV medications and was reviewed by the state Board of Pharmacy. After the medications were mailed to the peer organization, patients could pick up medications at the office, peers could provide transportation to the office, or peers could transport the medication package to the patient. Participants expressed appreciation for this assistance.

*The whole thing [medication locker storage] was helpful. I don’t think I would have been able to have done it without it. I didn’t have anywhere else to pick it up at or anything like that. The fact that they’d come and get me and bring me—I’d get my weekly amount—and take me back*,* that was real*,* real helpful. P6076*

*They mailed it to where my peer worker works. [My peer] would just have it mailed there to the office*,* then she would just deliver to me when it came. If I needed a refill*,* I would just call her. She would call it in*,* and then she would bring it to me when it came. That actually made it easier than me having to do the whole ordeal…She made everything way easier for me. P1201*

## Theme 3: Support addressing needs helped overcome barriers to care

Participants valued the instrumental support provided to overcome barriers and address needs, including: (a) assistance with resource and advocacy needs, and (b) proactive reminders and medication adherence support.

### Subtheme 3a. Assistance with resource and advocacy needs

Participants valued peers’ attentiveness to their daily living needs and support such as navigation to resources for housing, food, and other basic needs:

*Even if I needed help with anything else. Like if I needed support with anything else that was going on with my everyday life*,* anything as far as my household needs. Stuff like that*,* she was always real helpful with other things*,* gas support*,* with our roof leaking. She’d call to help us out with the resources*,* or give us phone numbers to reach out to. If she didn’t know*,* then she’d give us the resources or point us in the right direction…She went out of her way to help us out. She was really helpful. P1201*

Houselessness is a known barrier to HCV medication access and adherence [8]. In some cases, peers successfully used a study clinician letter and persistent advocacy to access Medicaid managed care organization funds for hotel stays to aid the patient in receiving, retaining, and adhering to the medication. In these instances, peers brought medication and other basic resources to the hotel.

*I got into a hotel because I didn’t want to take the medication until I got into a hotel. Because I live in the [encampment area]*,* which it’s pretty bad over there*,* and I was afraid somebody would steal my medication. I know it’s pretty expensive*,* so I didn’t want anybody taking my stuff. I left it here at [the peer office]*,* and then when I went and got the hotel*,* they brought me a four-week supply. Then I had to reapply or something like that for another four weeks… Carrying all my medication around with me if I didn’t [go to a hotel]*,* but that would have just been a pain in the ass. P1211*

*It was helpful because I didn’t have a vehicle to go pick it up. […] They dropped off the medication [at the hotel]*,* they dropped off food*,* they pretty much dropped anything off that I needed. P1211*

Hepatitis C medications are mailed by specialty pharmacies, some of which require a phone call with the patient to confirm mailing address and to ask about adherence prior to sending a medication refill. Peers and clinicians assisted with navigating this system barrier and intervened as necessary to ensure medications reached patients in a timely manner, as in the following example:

*I’m really frustrated that [the specialty pharmacy] were going to have me talk to the doctor again just to rewrite my prescription when all I did was wait for them to start it. They didn’t have to make it more complicated than it needed to be*,* and [my peer] had handled that. She asked to speak to the supervisor*,* and it was all handled. By the time I got off the phone with her*,* it was okay for me to go ahead…[My peer] was so great. P1255*

### Subtheme 3b. Proactive reminders and medication adherence support

Participants emphasized that receiving reminders from peers to adhere to their HCV medication regimen was essential. Peers reached out proactively to remind, encourage, and support participants in taking the medications.

*She would also help because*,* like I said*,* I don’t like taking pills [and] I would forget or something. She was very helpful with that. […] Because when she came and found me*,* she’s like*,* “You need to come with me. Come get your meds. Like*,* come on. P6157*

*[My peer] just made sure that I took the medication on time every day*,* and I checked in. If I needed questions asked or whatever*,* I would call them*,* and they were on top of it. They were very helpful. That’s pretty much it. It was all up to me whether or not I took the medication on time. I was pleased with the outcome*,* that’s for sure. P4053*

## Discussion

Our findings add to the literature on low-threshold care for PWUD that extends beyond traditional office-based approaches and engages people who experience structural barriers to care. Participants’ descriptions of their experience with the peer-assisted teleHCV model align with all aspects of low-threshold care as defined for MOUD: same-day treatment entry, harm reduction approach, flexibility (enabling patients to work around other obligations and needs), and availability in places frequented by people with OUD [21]. Participants’ descriptions of the model also aligned with many elements of low-threshold care as recently defined for HCV treatment [11], including flexibility, integrated peer support, providers’ values, addressing unmet social needs (including adapting medication delivery to patient needs), and care coordination. Similarly, Papalamprakopoulou et al.,(2025) found that participants valued the technical benefits of facilitated HCV telemedicine (convenience, overcoming transportation barriers) and the social value of the care provided (confidentiality and trust, empathetic clinicians, patient-centered care). Participants’ experiences in our study underscore how peer support and telemedicine-enabled care synergistically reduce stigma and facilitate treatment engagement and adherence. Although most accounts were strongly positive, a few participants described ongoing challenges, such as anxiety about blood draws, discomfort during phlebotomy despite staff efforts, or frustration with specialty pharmacy requirements, underscoring that even in a low-threshold model some aspects of care remained stressful.

### Staff approach and values facilitated engagement in care

Participants identified peers’ manner of engaging as a key aspect of the experience of care. We describe patient experience with peers in greater detail in a recent paper, including their ability to create an alliance with patients, role as a communication bridge with clinicians, and facilitation of access to resources [20]. Peers can play a key role in low-threshold care, bridging gaps in trust of the healthcare system due to past experiences, bringing a harm reduction approach, and addressing unmet basic needs. Providers also described peers as critical to the success of the model [19]. Past studies affirm that peers can promote emotional well-being [13, 14] and serve as intermediaries to help patients navigate the healthcare system [25]. Our findings suggest that peer support during blood draws eased anxieties and peer facilitation of telemedicine visits overcame digital barriers to access and bolstered confidence in engaging with healthcare. These observations are consistent with studies emphasizing the benefits of peer-facilitated telemedicine [45, 46].

Similarly, clinicians built rapport and trust through positive interactions, a critical feature for low-threshold care for PWUD [11] and person-first care [10, 15, 24]. Participants consistently reported positive and nonjudgmental interactions with study clinicians, contrasted with negative and stigmatizing previous experiences in healthcare settings. Peer and provider focus groups similarly highlighted the importance of provider approach and manner of engaging [19]. Studies using interviews with individuals with opioid use disorder have found that telemedicine can reduce the perception and experience of stigma encountered in conventional clinical settings [6, 40]. Telemedicine enables a vetted group of clinicians who use a patient centered approach to serve patients across broad geographic areas. Participants also appreciated lab technicians and phlebotomists who exhibited professionalism, kindness, and skill—essential for people with injection drug use history. Low-threshold models must attend to components outside the control of the team (such as phlebotomy), as these experiences can affect patients’ willingness to sustain engagement with the model. Peers and other study staff worked prior to study launch to identify and train supportive labs and phlebotomists, and peers provided support to patients during phlebotomy [20].

### Convenience and flexibility made care accessible

Participants identified key elements addressing where, when, and how care is delivered. This study highlights how low-threshold elements of the Peer TeleHCV model mitigated common barriers to care among rural, unstably housed, and marginalized patients. Peer and provider focus groups placed a similar value on these structural traits of the program [19]. Low-threshold models emphasize same-day treatment access, flexibility to work around patients’ needs, and availability in places frequented by people with substance use disorders (SUDs). Consistent with our findings, prior research has demonstrated that community-based, peer-facilitated telemedicine providing same-day access to care can improve treatment and medication engagement [45, 46]. The Peer TeleHCV model’s low-barrier approach, delivered through peers at convenient locations and times, overcame transportation and technology limitations and facilitated access. Supported medication delivery—including provision of secure medication storage—addressed well-documented challenges faced by people experiencing homelessness or unstable housing [3].

### Support addressing needs helped overcome barriers to care

Peers provided linkage to basic resources to meet daily needs, and both peers and clinicians provided system advocacy when needed. The model’s instrumental support aligns with elements of low-threshold treatment (addressing unmet social needs and care coordination) described by Finbråten et al., [11] and the support provided by case managers in the opioid treatment program-based model described by Papalamprakopoulou et al., [31]. Peers in our model also provided proactive support, reminders, and check-ins related to medication adherence.

### Limitations

This qualitative study has several limitations that should be considered when interpreting the findings. The study was conducted in rural Oregon, a setting with specific structural, policy, and health system contexts that may limit transferability to other geographic or policy environments. Findings are based on self-reported data, which may be influenced by social desirability bias and recall error. In addition, study staff were not able to reach some purposively sampled participants (lost to follow-up), which may have biased the sample toward those with more positive or complete experiences of the model. Perspectives of individuals who declined participation or were lost to follow-up were not captured and may have yielded more critical or ambivalent views. Although the team employed established methods to achieve analytic rigor, including double-coding and consensus review, qualitative interpretation inevitably reflects researcher positionality and context; the team’s experience in addiction medicine and harm reduction research may have influenced how they attended to and interpreted participants’ accounts. We did not conduct member checking by returning findings to participants, nor did we formally triangulate these interviews with other qualitative data sources, which may have limited opportunities to further validate our interpretations. Future research could consider perspectives from participants lost to follow-up and explore long-term outcomes of peer-facilitated telemedicine models.

## Conclusions

Our results provide patient perspectives on how peer-supported, telemedicine-enabled, community-embedded models advance care for PWUD. Community-based peers can engage people outside of care and provide access to mobile same-day telemedicine for hepatitis C, expanding equitable access to clinicians who use a harm reduction approach and provide care outside the traditional clinical context. This innovative low-threshold, community-centered model can foster trust in the healthcare system, address structural barriers, and forge pathways to care. The qualitative data from the EUC arm illustrate that without the streamlined, peer-facilitated telemedicine pathway, participants frequently did not engage with local HCV care, and only initiated treatment after being offered the Peer TeleHCV model — a finding consistent with the higher treatment initiation and cure rates observed in the TeleHCV arm of the parent trial [34].

While this study was conducted in rural Oregon, many contextual features that the model addressed—transportation barriers, limited access to specialty HCV care, and high rates of poverty and unstable housing—are common in other rural and underserved settings. For example, several core components of the Peer TeleHCV model are potentially adaptable to lower and middle income HCV elimination efforts: community-based peer outreach to engage people who use drugs outside of formal health services; case finding, navigation, and adherence support by trained peer workers; use of standing-order pretreatment laboratory workflows that can operate without clinician on-site review; and use of telemedicine to extend DAA prescribing into rural and underserved areas with clinician shortages. Our findings suggest that the core components of Peer TeleHCV—community-based peers, facilitated telemedicine with flexible scheduling, and adapted medication delivery—are likely transferable to similar low-resource environments, whereas aspects such as existing peer workforce infrastructure and partnerships with an academic medical center may be more site specific. Implementing similar models elsewhere will likely require intentional investment in building and sustaining a peer workforce (including training, supervision, and fair compensation), developing telehealth infrastructure that supports low-threshold access, and enacting policies and funding structures that recognize and support peer-delivered services outside the context of SUD treatment episodes. The model could be replicated in states that invest in supporting community-based peers and academic medical centers that provide telemedicine. Our study provides evidence that investment in low-threshold models engages a traditionally hard-to-reach population. On-demand telemedicine for buprenorphine is available in some states; adding infrastructure to support community-based peers can expand substance use and infectious disease care for people with SUDs. Innovative models like Peer TeleHCV can partner with state agencies to overcome structural and system barriers and scale and sustain implementation.

**Table 1:** Participant characteristics (*n* = 34)

Characteristic	Count (%)
Gender	
Female	14 (41%)
Male	20 (59%)
Age	
< 30	3 (9%)
30–39	10 (29%)
40–49	14 (41%)
50+	7 (21%)
Race or Ethnicity	
Hispanic/Latino	1 (3%)
Native American	3 (9%)
White Non-Hispanic	30 (91%)
Study Randomization Arm	
TeleHCV	22 (65%)
EUC	12 (35%))

## Data Availability

The data supporting this study’s findings are available from the corresponding author upon reasonable request, subject to participant confidentiality.
